# A simulation study to quantify the impacts of exposure measurement error on air pollution health risk estimates in copollutant time-series models

**DOI:** 10.1186/s12940-016-0186-0

**Published:** 2016-11-25

**Authors:** Kathie L. Dionisio, Howard H. Chang, Lisa K. Baxter

**Affiliations:** 1 National Exposure Research Laboratory, Office of Research and Development, U.S. Environmental Protection Agency, Research Triangle Park, NC USA; 2 Department of Biostatistics and Bioinformatics, Emory University, Atlanta, GA USA; 3 National Health and Environmental Effects Research Laboratory, Office of Research and Development, U.S. Environmental Protection Agency, Research Triangle Park, NC USA

**Keywords:** Exposure modeling, Exposure measurement error, Exposure assessment, Bias, Copollutant

## Abstract

**Background:**

Exposure measurement error in copollutant epidemiologic models has the potential to introduce bias in relative risk (RR) estimates. A simulation study was conducted using empirical data to quantify the impact of correlated measurement errors in time-series analyses of air pollution and health.

**Methods:**

ZIP-code level estimates of exposure for six pollutants (CO, NO_x_, EC, PM_2.5_, SO_4_, O_3_) from 1999 to 2002 in the Atlanta metropolitan area were used to calculate spatial, population (i.e. ambient versus personal), and total exposure measurement error.

Empirically determined covariance of pollutant concentration pairs and the associated measurement errors were used to simulate true exposure (exposure without error) from observed exposure. Daily emergency department visits for respiratory diseases were simulated using a Poisson time-series model with a main pollutant RR = 1.05 per interquartile range, and a null association for the copollutant (RR = 1). Monte Carlo experiments were used to evaluate the impacts of correlated exposure errors of different copollutant pairs.

**Results:**

Substantial attenuation of RRs due to exposure error was evident in nearly all copollutant pairs studied, ranging from 10 to 40% attenuation for spatial error, 3–85% for population error, and 31–85% for total error. When CO, NO_x_ or EC is the main pollutant, we demonstrated the possibility of false positives, specifically identifying significant, positive associations for copollutants based on the estimated type I error rate.

**Conclusions:**

The impact of exposure error must be considered when interpreting results of copollutant epidemiologic models, due to the possibility of attenuation of main pollutant RRs and the increased probability of false positives when measurement error is present.

**Electronic supplementary material:**

The online version of this article (doi:10.1186/s12940-016-0186-0) contains supplementary material, which is available to authorized users.

## Background

Many epidemiologic studies of the health effects associated with ambient air pollution exposure have focused on evaluating the effects associated with a single pollutant. In reality, humans are exposed to a complex mixture of pollutants that can vary spatially and temporally. The challenges of examining the relationship between multipollutant exposures and health effects include high correlations between pollutants preventing the use of standard statistical models, correlation of measurement errors across pollutants, varying degrees of exposure error across pollutants, the potential for interaction between pollutants, and pollutant mixtures varying by location [1–5]. Further, these epidemiologic studies often use data from centrally located measurement sites as the exposure estimate, which may not accurately capture the exposure variability due to the spatial variability of pollutant concentrations. As an example, analysis of pollutants with primarily local sources (CO, NO_x_, and EC) which exhibit a large degree of spatial heterogeneity [6–8] may be more error prone when central-site (CS) monitor measurements are used to estimate exposure, compared to pollutants primarily originating from regional sources (PM_2.5_, SO_4_, O_3_) which may exhibit spatially homogeneous patterns and thus have a lower degree of measurement error when CS monitor estimates are used. Fixed-location ambient monitors also do not account for human exposure factors such as where people move in time and space (e.g. in-vehicle concentrations impacting exposure), and concentrations in different microenvironments (e.g. infiltration-related factors). There is therefore the potential for exposure measurement error when CS measurements are used as a surrogate for exposure in epidemiologic studies. The presence of exposure measurement error can lead to effect attenuation and reduced statistical power in the resultant health risk estimate [9].

Billionnet et al. summarized the literature on statistical methods to study the effect of multiple pollutants [4], and Oakes et al. published a review of multipollutant exposure metrics [10]. Both identified a comprehensive understanding of exposure measurement error in the context of multipollutant studies as a key issue to address and investigate further. However while these papers detail work on multipollutant exposure metrics and statistical methods for analyzing multipollutant exposures, previous work to quantify the impact of exposure error on health risk estimates has focused on single-pollutant time-series models [6–8, 11–14]. While there are inherent difficulties in examining multipollutant exposures, we still do not have a clear understanding of the relationship of exposure measurement error in a more simplistic two pollutant model [15–17].

To our knowledge this is the first work which uses empirical pollutant relationships and health data to quantify the impact of correlated exposure measurement error in copollutant time-series models on resultant health risk estimates in a simulation study. We consider Poisson regression and allow for additive, multiplicative, and correlated measurement errors, which are typically not considered in the classical/Berkson error framework [16]. We use previously described, daily exposure metrics ranging from CS measurements to more complex modeling approaches [18] to calculate empirical relationships between pollutants (PM_2.5_, SO_4_, O_3_, CO, NO_x_, EC). Relationships between exposure metrics were previously used to calculate estimates of exposure measurement error due to spatial variability of pollutant concentrations, and human exposure factors at the ZIP code level [17], but did not include the use of empirical health data nor analysis of an epidemiological model. Our previous work showed the potential for bias in model coefficients for copollutant models, motivating the current work to examine the degree of attenuation of relative risks (RRs) empirically. In the current paper, the previous work is extended in a simulation study by considering a Poisson time-series analysis of emergency department (ED) visits for respiratory disease in the Atlanta, GA metropolitan area to obtain empirical estimates of the attenuation in health risk estimates due to various sources of exposure measurement error.

## Methods

### Estimates of exposure and exposure measurement error

Three estimates of daily exposure to ambient PM_2.5_, EC, SO_4_, CO, NO_x_, and O_3_ were derived for 193 ZIP codes in the 20-county Atlanta metropolitan area. Pollutant-specific measured concentrations, modeled exposure estimates, and summary statistics have been described previously in detail [17, 18]. Briefly the three exposure assessment approaches include: (1) CS measurements (CS), (2) ambient air quality (AQ) estimates obtained by combining simulations from an air quality model for regional background and a dispersion model for the local contribution, and (3) estimates from a stochastic population exposure model (PE). For PE, the contribution from indoor sources was not included because of the desire to examine the association between the health outcomes and exposure to ambient pollution, and for comparability with the CS measurements and AQ estimates. All three approaches estimate exposures to ambient pollution at each ZIP code centroid in the study area. Daily estimates (8-h maximum for O_3_, 24-h average for other pollutants) for 1999–2002 were generated for each approach.

For each ZIP code, three types of exposure error (δ_spatial_, δ_population_, δ_total_) were calculated as the difference between two exposure metrics. Detailed summary statistics including the magnitude and variance of δ, and between-pollutant correlations of δ are presented in [17]. Briefly, the ZIP code-specific exposure error due to a lack of spatial refinement in the exposure estimate is represented by δ_spatial_ = AQ - CS, assuming that the difference between the more (i.e. AQ) and less (i.e. CS) spatially refined metrics gives an estimate of the amount of spatial measurement error that is present in the CS metric. This assumption is made because our air quality models add spatial variability to the AQ metric compared with CS measurements, which lack spatial variability because the same CS measurement was used to represent exposure in each ZIP code. Exposure error introduced when human exposure factors are not included in an exposure estimate is represented by δ_population_ = PE – AQ. Our PE metric includes variability due to human exposure factors such as time-location-activity patterns of individuals, commuting patterns, and infiltration of ambient pollutants to indoor environments. A third type of exposure error, δ_total_ = PE – CS, represents the combined exposure error when both spatial variability and human exposure factors are not accounted for. δ_total_ does not represent all potential sources of exposure error that may be present in a study; instead it represents the total exposure error that we were able to assess in this analysis.

### Epidemiologic model

To conduct the simulation, health data from a previously described epidemiologic study were used [19]. Briefly, individual-level data on ED visits for respiratory outcomes from 41 hospitals in the 20-county Atlanta area were aggregated to daily counts for each ZIP code. The same Poisson time-series model in S. Sarnat et al. [19] was used in this simulation study to examine the association between the above described exposure metrics and daily counts of ED visits. The basic form of the model was:

1 $$ \begin{array}{l} \log \left(E\left({Y}_{kt}\right)\right)=\propto +{\beta}_1 pollutio{n}_{1,kt}+{\beta}_2 pollutio{n}_{2,kt}+\\ {}{{\displaystyle \sum}}_k{\lambda}_kZI{P}_k+{{\displaystyle \sum}}_m{\lambda}_m DO{W}_{mt}+{{\displaystyle \sum}}_n{\nu}_n hospita{l}_{nt}+\\ {}g\left({\gamma}_1,\dots, {\gamma}_N;tim{e}_t\right)+{{\displaystyle \sum}}_o{\xi}_o IOtem{p}_{ot}+\\ {}{\eta}_1 dewp{t}_t+{\eta}_2 dewp{t}_t^2+{\eta}_3 dewp{t}_t^3+\\ {}{\delta}_1tem{p}_t+{\delta}_2tem{p}_t^2+{\delta}_3tem{p}_t^3\end{array} $$

where *Y*_*kt*_ is the count of ED visits for respiratory outcomes in ZIP code k on day t. For each pollutant (*pollution*_*1*_ *and pollution*_*2*_), daily averages (8-h maximum for O_3_, 24-h average for all other pollutants) of same-day concentrations were used. To control for spatial autocorrelation in the baseline ED visits across the ZIP codes, an indicator variable for the k^th^ ZIP code (*ZIP*_*k*_) was used to represent the areas from which ED counts were spatially aggregated. Dummy variables for day of week and holidays (*DOW, *indexed by *m*) and for hospital (*hospital,* indexed by *n*) were used; the latter accounted for the differing durations of time each hospital was included in the study. Long-term trends and seasonality of health outcomes (*time*) were controlled for with parametric cubic splines with monthly knots (*g*(*γ*_1_, …, *γ*_*N*_; *x*)). Meteorology was controlled for using maximum temperature (*IO*_*temp*_) with indicators for each degree Celsius, a cubic function for dew point (*dewpt*), a cubic function for minimum temperature (*temp*), and dummy variables for seasons. When simulating the health effect we assume zero lag, but expect results to be generalizable to single day lags.

### Simulation of true exposures and health outcomes

True exposure was defined as an exposure estimate without spatial and/or population measurement error. To simulate the true daily exposure for each pollutant, linear models for each type of daily exposure were fitted separately for each ZIP code and for each pollutant; for example, for spatial measurement error, the model is given by *AQ*_*kt*_ = *θ*_*k*,1_ + *θ*_*k*,2_ * *CS*_*kt*_ + *ε*_*k*,*t*_ for ZIP code k and day t, such that both additive (*θ*_1_) and multiplicative bias (*θ*_2_) between the two exposure metrics were accounted for. See Additional file 1: Table S1 for the mean and standard deviation across ZIP codes of estimates of θ _k,1_ and θ _k,2_. For each Monte Carlo iteration, we first calculated a ‘true’ exposure mean for each day by using the unrefined measurement as the predictor (i.e., CS for spatial or total measurement error, AQ for population measurement error) and a realization of the measurement error coefficients (*θ*_1_ and *θ*_2_) drawn from their asymptotic bivariate normal distribution. Across-ZIP code spatial heterogeneity in the measurement error coefficients was allowed, however for each ZIP code we assumed that additive and multiplicative bias remains constant across days. Finally, the residual of the true exposure (*ε*_*t*_) was subsequently drawn from a bivariate normal distribution with covariance equal to the ZIP code specific empirical measurement error covariance (see Additional file 1: Table S2 for a summary of the median across ZIP codes of the correlation of measurement error covariance) for the two pollutants of interest, and then combined with the true exposure mean to obtain the full true exposure value (*true_exp*). We chose to simulate true exposure from the less refined exposure because our measurement error framework assumes that the true exposure is more variable than the error-prone (unrefined) exposure. The resultant measurement error between the true and error-prone exposures, derived here from empirical data, do not strictly follow the standard classical or Berkson measurement error models (Zeger et al. [16]).

Using observed health data from the study described in S. Sarnat et al. [19], a design matrix $X$ (without the two pollutants) was constructed for the Poisson model in Equation (1). The Poisson mean $\mu$ for each day and each ZIP code was calculated as:

2 $$ \log \left(\mu \right)=X\times B+ \log R{R}_1\ \frac{true\_ ex{p}_1}{IQ{R}_1}+ \log R{R}_2\ \frac{true\_ ex{p}_2}{IQ{R}_2} $$

where *B* is a vector of the estimated regression coefficients from Equation (1), subscripts 1 and 2 denote pollutants 1 and 2, *RR*_1_ and *RR*_2_ are the hypothetical RRs per interquartile range (IQR) increase to allow for comparison across pollutants, *true_exp*_1_ and *true_exp*_2_ are the simulated true exposures, and *IQR*_1_ and *IQR*_2_ are the interquartile ranges for the corresponding true exposure metrics. We assumed *RR*_1_ = 1.05 and *RR*_2_ = 1, and in subsequent text refer to pollutant 1 as the ‘main pollutant’ (i.e., the pollutant with an assumed health effect) and to pollutant 2 as the ‘copollutant’ (i.e., the pollutant assumed to have no effect). The assumed *RR*_1_ = 1.05 was chosen based on previously established single-pollutant models for the same data set, specifically the O_3_-respiratory analysis [19]. Poisson counts were drawn from a Poisson distribution of the simulated Poisson mean *μ*. Two versions of the Poisson time-series model (Equation (1)) were then fitted: one using the simulated Poisson counts with the true exposure, and another using the same simulated Poisson counts, together with the unrefined exposure values (standardized by their corresponding IQRs). This resulted in two sets of estimated log RR: ${\widehat{\beta}}_{1,\  true}$ and ${\widehat{\beta}}_{2,\  true}$ for the ‘true exposure’ scenario, and ${\widehat{\beta}}_{1,\  noisy}$ and ${\widehat{\beta}}_{2,\  noisy}$ for the ‘noisy exposure’ scenario (i.e., using the unrefined exposure metric).

The above simulation was run 1000 times for each pollutant pair and measurement error scenario. The mean over the 1000 estimates for each RR was calculated and used as the overall point-estimate associated with each pollutant pair and measurement error scenario. As an example, the RR for the main pollutant, true exposure scenario, was calculated as:

3 $$ {\overline{RR}}_{1,\  true}= \exp \left(\frac{{\displaystyle {\sum}_{n=1}^N}{\widehat{\beta}}_{1,\  true,\ n}}{N}\right) $$

where n indexes the simulation iteration and *N* = 1000. Standard error of the RR was calculated as the standard error of the 1000 *β*s. A student’s two-sided *t*-test was used to determine significant differences between ${\overline{RR}}_{noisy,a}$ and ${\overline{RR}}_{noisy,b}$ for two different copollutants *a* and *b*, when paired with the same main pollutant, across Monte Carlo simulations.

### Statistical analyses

Mean RR estimates from using the true and noisy exposures were compared to the true RR in order to determine if the presence of measurement error induces bias and a loss of power. Various summary statistics are presented, including percent attenuation, root mean-square error (RMSE), and power/type I error.

Percent attenuation of the RR for the main pollutant was calculated to determine the impact of inclusion of measurement error on attenuation of the RR. RMSE was calculated to assess both bias and loss of precision in the estimates. Lastly, statistical power/type I error was calculated to assess false positive associations. As an example, for pollutant 1, noisy scenario, we define:

4 $$ \%\  attenuatio{n}_{1, noisy} = \frac{R{R}_1 - \overline{R{R}_{1,\  noisy}}}{R{R}_1-1} $$

5 $$ RMS{E}_{1, noisy}=\sqrt{\frac{{\displaystyle {\sum}_{n=1}^N}{\left({\widehat{\beta}}_{1, noisy,\ n} - \log R{R}_1\right)}^2}{N}} $$

6 $$ Powe{r}_{1, noisy} = \mathrm{\mathbb{P}}\left(\frac{{\widehat{\beta}}_{1,\  noisy}}{s_{\beta_{1, noisy}}}>1.96\right) $$

7 $$ S{E}_{Powe{r}_1, noisy} = \sqrt{\frac{Powe{r}_{1, noisy}*\left(1- Powe{r}_{1, noisy}\right)}{N}} $$

where *RR*_1_ = 1.05, *RR* = 1, *N* = 1000, *β* and *s*_*β*_ (standard error of *β*) are obtained from the Poisson model (equation 1), and ${\widehat{RR}}_{1,\  noisy}$ is defined as in equation 3. Quantities were similarly calculated for each pollutant pair and measurement error scenario. RMSE ratio was defined as *RMSE*_*noisy*_/*RMSE*_*true*_ for pollutant 1 or 2.

All statistical analyses and model simulations were completed in R, version 3.0.2 (R Foundation for Statistical Computing; http://www.r-project.org/).

## Results

**Fig. 1:**
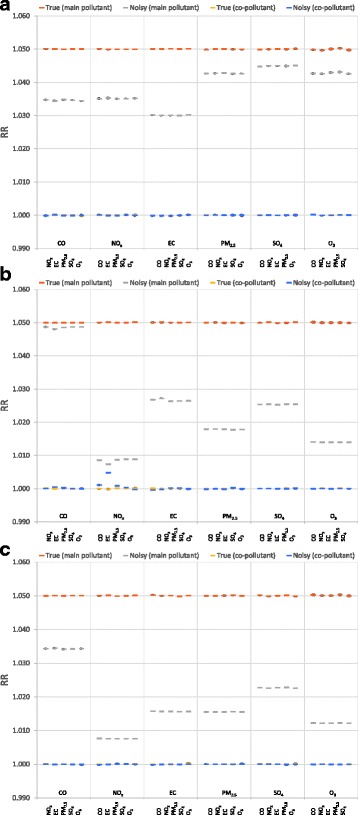
Attenuation of RR due to measurement error in a copollutant model (RR_1_ = 1.05, RR_2_ = 1). For x-axis labels, top row indicates the main pollutant (pollutant 1), bottom row indicates the copollutant (pollutant 2). Overall point estimates shown are the mean over 1000 estimates; error bars indicate the 95^th^ confidence interval for the 1000 estimates (i.e., the 2.5^th^ and 97.5^th^ percentiles of simulated effect estimates; note that extremely narrow confidence intervals result in some non-visible error bars). Note that when copollutant true and noisy model results do not differ substantially, plotted data points overlap. **a** Spatial measurement error (δ_spatial_). **b** Population measurement error (δ_population_). **c** Total measurement error (δ_total_)

Mean RRs for the main pollutant over 1000 runs of the health model for both the noisy and true exposure metrics are shown in Fig. 1. For all types of measurement error, resultant RR of the main pollutants (pollutant 1) when the health model used the true exposure metric (i.e., the simulated exposure metric without measurement error) is close to 1.05. Similarly, the resultant RR of the copollutants (pollutant 2) using the true exposure metric is close to 1, as expected due to the assumed *RR*_1_ = 1.05 and *RR*_2_ = 1. In contrast the presence of measurement error in the noisy exposure metric results in considerable attenuation for all types of measurement error (Fig. 1, Table 1). For spatial measurement error, we see greater attenuation for ${\overline{RR}}_{1, noisy}$ when a local pollutant (CO, NO_x_, EC) is the main pollutant (29–40%) compared to a regional pollutant (PM_2.5_, SO_4_, O_3_; 10–15%) (ranges represent the attenuation across all copollutants models for a specific main pollutant). For population measurement error, there is little attenuation when CO is the main pollutant (3–4%), and substantial attenuation (82–85%) of ${\overline{RR}}_{1, noisy}$ when NO_x_ is the main pollutant. Similarly for total measurement error, the highest level of attenuation of ${\overline{RR}}_{1, noisy}$ is seen when NO_x_ is the main pollutant (85%), and the least attenuation for CO (31–32%). For all other pollutants, for both population and total measurement error, attenuation of ${\overline{RR}}_{1, noisy}$ is moderate-high (Table 1). For all pollutants and all types of measurement error, attenuation of ${\overline{RR}}_{1, noisy}$ mirrors the patterns of attenuation hypothesized in previous analyses (see figure four of Dionisio et al., 2014) [17] . The scenario with an assumed *RR*_1_ = 1.05 and *RR*_2_ = 1.05 was run as a sensitivity analysis. Resultant RR from the sensitivity analysis are presented in Additional file 1: Figure S1. Two-sided t-tests were used to compare differences in attenuation of the main pollutant RR by copollutant; there were minimal differences seen across copollutants for the case of *RR*_1_ = 1.05 and *RR*_2_ = 1, with more differences seen for the sensitivity analysis (see Additional file 1: Supplemental Text, Table S3, and Table S4). The coverage of the 95% confidence intervals was calculated for the main pollutant and copollutant for each of the different scenarios analyzed, and with one exception was in the range of 0.93–0.98.

**Table 1:** Percent attenuation of RR_1,noisy_ by main pollutant and measurement error type

Main pollutant	δ_spatial_	δ_population_	δ_total_
Local			
CO	30–31%	3–4%	31–32%
NO_x_	29–30%	82–85%	85%
EC	40%	46–47%	69%
Regional			
PM_2.5_	14–15%	64–65%	69%
SO_4_	10%	49%	54–55%
O_3_	14–15%	72%	75–76%

The RMSE ratios comparing the RMSE for the noisy and true model estimates are presented in Table 2, reflecting both bias and loss of precision. The RMSE ratio for the copollutants is typically small (≤2.0), while the RMSE ratio for the main pollutants ranges from 1.3 to 26.1. While the main pollutant RMSE ratio for regional pollutants (PM_2.5_, SO_4_, O_3_) ranges from 1.8 to 13.7, there is little difference seen across copollutants and measurement error type for the same main pollutant. In contrast, the copollutant can have a substantial impact on the main pollutant RMSE ratio when local pollutants (CO, NO_x_, EC) are the main pollutant. For example, for δ_spatial_ for CO, NO_x_, and EC as the main pollutant, the RMSE ratio of the main pollutant ranges from 6.6–11.5, 6.0–10.4, and 6.6 – 12.1 respectively, dependent upon the copollutant (Table 2).

**Table 2:** RMSE ratios from comparison of bipollutant models (RR_1_ = 1.05, RR_2_ = 1) with and without measurement error

		δ_spatial_		δ_population_		δ_total_	
Main pol.^a^	Co-pol.^a^	Main pol.	Co-pol.	Main pol.	Co-pol.	Main pol.	Co-pol.
CO	NO_x_	6.6	1.9	1.9	0.4	8.0	0.5
	EC	8.2	1.3	1.3	0.6	8.1	0.7
	PM_2.5_	11.1	1.4	1.5	0.4	10.9	0.5
	SO_4_	11.0	1.2	1.4	0.5	10.9	0.6
	O_3_	11.5	1.2	1.5	0.3	12.2	0.4
NO_x_	CO	6.0	1.8	13.7	2.0	16.9	2.0
	EC	6.1	1.1	13.3	1.6	16.1	0.6
	PM_2.5_	9.1	1.2	23.6	0.5	23.4	0.5
	SO_4_	10.3	1.1	25.6	0.6	24.1	0.6
	O_3_	10.4	1.1	25.8	0.3	26.1	0.4
EC	CO	7.9	1.8	6.6	1.1	13.3	1.7
	NO_x_	6.6	1.6	6.6	0.3	13.3	0.4
	PM_2.5_	10.3	1.3	11.4	0.4	17.4	0.5
	SO_4_	11.4	1.1	14.0	0.6	18.0	0.6
	O_3_	12.1	1.1	13.5	0.3	19.0	0.4
PM_2.5_	CO	2.9	1.8	11.4	1.0	13.5	1.9
	NO_x_	2.9	1.8	11.1	0.2	13.2	0.4
	EC	2.7	1.3	10.4	0.6	12.4	0.8
	SO_4_	2.5	1.2	9.4	0.5	10.7	0.7
	O_3_	2.9	1.0	12.1	0.3	13.7	0.3
SO_4_	CO	2.1	1.7	9.6	1.0	10.5	1.6
	NO_x_	2.0	1.6	9.6	0.2	10.6	0.4
	EC	2.1	1.2	9.4	0.6	10.0	0.7
	PM_2.5_	1.9	1.2	6.8	0.4	8.4	0.5
	O_3_	2.0	1.0	9.0	0.3	10.1	0.3
O_3_	CO	1.8	1.6	8.2	1.0	8.3	1.6
	NO_x_	1.9	1.8	8.5	0.2	8.9	0.3
	EC	1.8	1.1	8.3	0.6	8.6	0.6
	PM_2.5_	1.8	1.1	8.1	0.4	8.8	0.4
	SO_4_	1.8	1.0	7.8	0.6	8.0	0.5

^a^In simulations, all main pollutants had RR = 1.05, and all co-pollutants had RR = 1. For each pollutant pair, 1000 simulations were run and results averaged

**Fig. 2:**
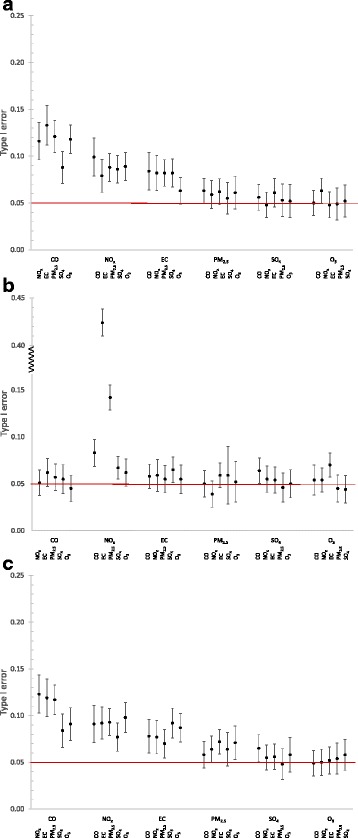
Type I error for the copollutant (pollutant 2) in a copollutant model (RR_1_ = 1.05, RR_2_ = 1), with exposure measurement error. For x-axis labels, top row indicates the main pollutant (pollutant 1), bottom row indicates the copollutant (pollutant 2). Red line indicates type I error = 0.05. Overall point estimates shown are the mean over 1000 estimates; error bars indicate the 95^th^ confidence interval for the 1000 estimates. **a** Spatial measurement error (δ_spatial_). **b** Population measurement error (δ_population_). **c** Total measurement error (δ_total_)

Power estimates for all main pollutants were equal to 1, indicating we will always detect the health effect association for the main pollutant (results not shown) given the simulation setup. Copollutant type I error for the true scenarios (i.e., no measurement error) were approximately 0.05 (results not shown). We present the type I error for the copollutant, noisy scenario (i.e., with measurement error), when the copollutant has no effect (RR_2_ = 1) (Fig. 2). Figure 2 shows that for CO, NO_x_, or EC as the main pollutant there is a substantial increase in the type I error of the copollutant when spatial or total exposure measurement error is present in both the main pollutant and the copollutant, increasing the likelihood of detecting false positive associations for the copollutants when CO, NO_x_ or EC are the main pollutant in these instances. For copollutants paired with NO_x_ under the δ_population_ scenario we see larger type I error values, particularly for NO_x_ paired with EC. For PM_2.5_, SO_4_, and O_3_ as the main pollutant for δ_spatial_ and δ_total_ and for all pollutants except NO_x_ for δ_population_, there is less likelihood of detecting false positive associations.

## Discussion

While previous work has examined the impact of exposure measurement error in single pollutant models [13] and studies have proposed statistical methods for handling multipollutant effects [15, 20–22], little work has been done to quantify the impact of measurement error on health risk estimates in multipollutant models, including copollutant models. This study builds on previous work examining the potential attenuation of model coefficients in copollutant epidemiologic models based on empirical covariance structures [17]. While the previous work built the foundation for the current analysis through development of exposure metrics and calculation of empirical relationships between pollutants, the current manuscript extends the work with the addition of empirical health data applied in an epidemiological model with the previously developed exposure metrics. Using the empirical measurement error covariance structures, estimates of additive and multiplicative bias between exposures, and between-pollutant relationships, we extended the previous work by conducting a simulation study to assess the impact of measurement error on health risk estimates obtained from a copollutant time-series model. We show the substantial attenuation of RRs resulting from the presence of exposure measurement error in exposure estimates for most pollutants and measurement error types examined, an empirical result which agrees with the predictions in previous work [17]. Lastly, we have shown evidence for the detection of false positive associations between the copollutant and the health outcome, particularly when CO, NO_x_, or EC are the main pollutant.

The RMSE ratio comparing the RMSE of the risk estimates from the noisy and true exposures reflects both the bias and standard error of the estimates. When we compare the RMSE ratio for a main pollutant-measurement error type, we see that for regional pollutants as the main pollutant, RMSE ratio changes very little across copollutants. However the RMSE ratio of the local pollutants as main pollutants is impacted substantially depending upon whether regional or local pollutants are the copollutants. For all local pollutant- (as main pollutant) measurement error pairs except for CO-population measurement error, we see a substantial increase in RMSE ratio for the main pollutant when the copollutant is a regional pollutant, compared to when the copollutant is a local pollutant. Thus from examination of the RMSEs (Table 2) we conclude that the inclusion of a regional copollutant in a model with a local main pollutant substantially increases the degree of bias and standard error of the main pollutant estimates, compared to having a local copollutant. However Fig. 1 demonstrates that the copollutant (whether regional or local) has little effect on the degree of bias in the main pollutant’s effect estimate. The two results taken together allow us to conclude that the differences in RMSE across copollutant type are due to differences in standard error, rather than bias, with the spatial structure of the copollutant influencing the standard error but not the bias of the main pollutant’s effect estimate. We also see differences in the RMSE ratio of local pollutants as main pollutants when the copollutant is varied, even when the copollutants have an assumed null effect (RR = 1). This indicates that relationship of these copollutants and their error with the main pollutant does have an impact on the bias and standard error of main pollutant RR estimates.

Type I error estimates in this study show the likelihood of detecting a false positive effect for a copollutant with an assumed null effect (i.e., RR = 1) is often reduced when measurement error is not present, especially when CO, NO_x_, and EC are the main pollutants. It is likely that this impact is more evident for CO, NO_x_, and EC than it is for PM_2.5_, SO_4_, and O_3_ because the former are dominated by local sources, thus tend to be more spatially variable [17] and have higher degrees of correlated exposure measurement error. This can be important when trying to understand the independent effect of a pollutant that is a part of a complex mixture. For example, the EPA’s most recent Integrated Science Assessments (ISAs) for CO [23] and NO_x_ [24] both state that while there is evidence of consistent positive associations between short-term exposure to CO and NO_2_ and effects on the respiratory system in epidemiologic studies, the challenge remains to disentangle the independent effect of CO or NO_2_ related health effects from the larger air pollution mixture. There is the possibility that CO and/or NO_x_ are serving as indicators of combustion-related emissions, particularly from traffic, for some health outcomes. The moderate to high correlations between CO, NO_x_, and other pollutants generated from combustion processes (e.g., EC, PM_2.5_) further complicates interpretation of epidemiologic studies of these pollutants. This provides motivation for the use of exposure metrics which minimize measurement error in epidemiologic analyses.

While the simulation presented here has many strengths, including accounting for multiplicative bias and correlated measurement errors, and the use of empirical data, there are inherent limitations. First, we assume that the refined exposure metric in each of our three constructed measurement error types provides an accurate representation of measurement error. That is, we assume that the AQ exposure metric accurately and completely accounts for spatial variability ignored in the CS metric, and we assume the PE exposure metric appropriately accounts for population variability ignored in the AQ metric. The authors acknowledge that the AQ metric and PE metrics themselves include some error, but in the absence of a ‘true’ spatially refined metric (e.g., a fine-scale measurement/monitor network in the study area) and a set of population-based personal exposure measurements, we operate under the above assumptions. Any error present in the refined metrics will remain an influence on the calculated RRs. Further, though it is common to use an individual’s exposure to ambient-generated pollution in an epidemiologic study, the inclusion of exposure from indoor sources would allow for analysis of the impact of exclusion of this exposure source on commonly calculated RRs. Though we expect results presented here to have general applicability regarding the impact on bias of RRs in a copollutant time-series model, we acknowledge that results presented here are dependent on empirical data from the Atlanta metropolitan area. Multipollutant relationships may be different in other cities depending on the city-specific source profile and the magnitude of measurement errors may depend on the location of central monitor and population characteristics.

Simulation studies have examined the impact of measurement error in hypothetical multipollutant epidemiologic studies [16, 25]. Exposure measurement error is often categorized into two classes: Berkson error and classical error, which are often assumed to be independent of the true exposure [16]. In reality, exposure measurement error is a combination of the two. Adding to the complexity, the consequences of each error type are different [5, 26]. In most time-series studies, Berkson error will not bias the effect estimates, but will tend to increase the variance of the regression coefficients (increasing the width of the CIs and decreasing power), while classical measurement error tends to bias the true effect towards the null, with the magnitude of the effect attenuation depending on the error variance of the exposure estimate relative to the variance of the true exposure [16]. Empirical analyses have shown impacts of both types of error in single pollutant models [14, 27–29]. When two pollutants are measured with error, the correlation between the pollutants, and the correlation between their errors, predicts the magnitude of the bias, while the sign of the correlation between the pollutants predicts the direction of the bias [16, 20]. In this analysis, we do not follow standard classical or Berkson measurement error models, but utilize the empirical relationships between different exposure metrics from a real-world epidemiologic study. By assuming that the more refined exposure metric is the true exposure while treating the less refined metric as the error-prone exposure, we observe effect attenuation and bias away from the null, depending on the copollutant pair considered. In addition, we note that time-series analyses typically rely on a small number of monitors to derive population-averaged exposure metrics. Recently there has been increasing interest in conducting spatio-temporal modeling of air pollutants via land-use regression models [30] and data fusion methods [31] to capture spatial heterogeneity within the study region. However, measurement errors are also associated with spatial predictions due to spatial smoothing and estimation uncertainty [21, 32].

## Conclusions

To the best knowledge of the authors, this study is the first to quantify the impact of measurement error on health risk estimates in copollutant models using empirical data. In addition, this study directly addresses a question often considered when examining the collective body of evidence with respect to copollutant models: the impact of varying degrees of exposure measurement error on resultant risk estimates. Based on results presented here, the impact of measurement error in future studies of the health effects of exposure to air pollution must be considered, so that the true health impact of air pollution exposure is not underestimated, and to avoid false conclusions. In addition, future studies may investigate additional techniques and statistical methods for measurement error correction.

## Additional file

Additional file 1: Supplemental materials including supplemental text, figures, and tables, are available as an additional file. (PDF 392 kb)

## Abbreviations

AQ: Ambient air quality estimates
CS: Central-site measurements
ED: Emergency department
ISA: Integrated science assessment
PE: Population exposure model estimates
RMSE: Root mean-square error
RR: Relative risk

## References

[1] Dominici F, Peng RD, Barr CD, Bell ML (2010). Protecting human health from Air pollution: shifting from a single-pollutant to a multipollutant approach. Epidemiology.

[2] Mauderly JL, Burnett RT, Castillejos M, Özkaynak H, Samet JM, Stieb DM, Vedal S, Wyzga RE (2010). Is the air pollution health research community prepared to support a multipollutant air quality management framework?. Inhal Toxicol.

[3] Vedal S, Kaufman JD (2011). What does multi-pollutant air pollution research mean?. Am J Respir Crit Care Med.

[4] Billionnet C, Sherrill D, Annesi-Maesano I (2012). Study ObotG: estimating the health effects of exposure to multi-pollutant mixture. Ann Epidemiol.

[5] Bateson TF, Coull BA, Hubbell B, Ito K, Jerrett M, Lumley T, Thomas D, Vedal S, Ross M (2007). Panel discussion review: session three - issues involved in interpretation of epidemiologic analyses - statistical modeling. J Expo Sci Environ Epidemiol.

[6] Goldman GT, Mulholland JA, Russell AG, Srivastava A, Strickland MJ, Klein M, Waller LA, Tolbert PE, Edgerton ES (2010). Ambient Air pollutant measurement error: characterization and impacts in a time-series epidemiologic study in Atlanta. Environ Sci Technol.

[7] Sarnat SE, Klein M, Sarnat JA, Flanders WD, Waller LA, Mulholland JA, Russell AG, Tolbert PE (2010). An examination of exposure measurement error from air pollutant spatial variability in time-series studies. J Expo Sci Environ Epidemiol.

[8] Strickland MJ, Gass KM, Goldman GT, Mulholland JA (2015). Effects of ambient air pollution measurement error on health effect estimates in time-series studies: a simulation-based analysis. J Expo Sci Environ Epidemiol.

[9] Shy CM, Kleinbaum DG, Morgenstern H (1978). The effect of misclassification of exposure status in epidemiological studies of air pollution health effects. Bull NY Acad Med.

[10] Oakes M, Baxter L, Long T (2014). Evaluating the application of multipollutant exposure metrics in air pollution health studies. Environ Int.

[11] Setton E, Marshall JD, Brauer M, Lundquist KR, Hystad P, Keller P, Cloutier-Fisher D (2011). The impact of daily mobility on exposure to traffic-related air pollution and health effect estimates. J Expo Sci Environ Epidemiol.

[12] Goldman GT, Mulholland JA, Russell AG, Strickland MJ, Klein M, Waller LA, Tolbert PE (2011). Impact of exposure measurement error in air pollution epidemiology: effect of error type in time-series studies. Environ Health.

[13] Dominici F, Zeger SL, Samet JM (2000). A measurement error model for time-series studies of air pollution and mortality. Biostatistics.

[14] Hart JE, Liao X, Hong B, Puett RC, Yanosky JD, Suh H, Kioumourtzoglou M-A, Spiegelman D, Laden F (2015). The association of long-term exposure to PM_2.5_ on all-cause mortality in the Nurses’ Health Study and the impact of measurement-error correction. Environ Health.

[15] Chang HH, Peng RD, Dominici F (2011). Estimating the acute health effects of coarse particulate matter accounting for exposure measurement error. Biostatistics.

[16] Zeger SL, Thomas D, Dominici F, Samet JM, Schwartz J, Dockery D, Cohen A (2000). Exposure measurement error in time-series studies of air pollution: concepts and consequences. Environ Health Perspect.

[17] Dionisio KL, Baxter LK, Chang HH (2014). An empirical assessment of exposure measurement error and effect attenuation in bipollutant epidemiologic models. Environ Health Perspect.

[18] Dionisio KL, Isakov V, Baxter L, Sarnat JA, Sarnat SE, Burke J, Graham SE, Mulholland J, Özkaynak H (2013). Development and evaluation of alternative approaches for exposure assessment of multiple air pollutants in Atlanta, Georgia. J Expo Sci Environ Epidemiol.

[19] Sarnat SE, Sarnat JA, Mulholland J, Isakov V, Özkaynak H, Chang H, Klein M, Tolbert PE (2013). Application of alternative spatiotemporal metrics of ambient air pollution exposure in a time-series epidemiological study in Atlanta. J Expo Sci Environ Epidemiol.

[20] Zeka A, Schwartz J (2004). Estimating the independent effects of multiple pollutants in the presence of measurement error: an application of a measurement-error-resistant technique. Environ Health Perspect.

[21] Bergen S, Sheppard L, Kaufman JD, Szpiro AA. Multipollutant measurement error in air pollution epidemiology studies arising from predicting exposures with penalized regression splines. J R Stat Soc: Ser C: Appl Stat. 2016;65(5):731–53. 10.1111/rssc.12144 PMC5076926 27789915

[22] Schwartz J, Coull BA (2003). Control for confounding in the presence of measurement error in hierarchical models. Biostatistics.

[23] U.S. EPA (2010). Integrated science assessment for carbon monoxide. vol. EPA/600/R-09/019F.

[24] EPA US (2008). Integrated science assessment for oxides of nitrogen-health criteria. vol. EPA/600/R-08/071.

[25] Zidek JV, Wong H, Le ND, Burnett R (1996). Causality, measurement error and multicollinearity in epidemiology. Environmetrics.

[26] Thomas D, Stram D, Dwyer J (1993). Exposure measurement error: influence on exposure-disease relationships and methods of correction. Annu Rev Public Health.

[27] Hart JE, Spiegelman D, Beelen R, Hoek G, Brunekreef B, Schouten LJ, Brandt Pvd. Long-Term Ambient Residential Traffic-Related Exposures and Measurement Error-Adjusted Risk of Incident Lung Cancer in the Netherlands Cohort Study on Diet and Cancer. Environ Health Perspect. 2015;123(9):860–66. 10.1289/ehp.1408762 PMC4559954 25816363

[28] Van Roosbroeck S, Li R, Hoek G, Lebret E, Brunekreef B, Spiegelman D (2008). Traffic-related outdoor air pollution and respiratory symptoms in children: the impact of adjustment for exposure measurement error. Epidemiology.

[29] Li R, Weller E, Dockery DW, Neas LM, Spiegelman D (2006). Association of indoor nitrogen dioxide with respiratory symptoms in children: application of measurement error correction techniques to utilize data from multiple surrogates. J Expo Sci Environ Epidemiol.

[30] Hu X, Waller LA, Lyapustin A, Wang Y, Al-Hamdan MZ, Crosson WL, Estes MG, Estes SM, Quattrochi DA, Puttaswamy SJ, Liu Y (2014). Estimating ground-level PM2.5 concentrations in the Southeastern United States using MAIAC AOD retrievals and a two-stage model. Remote Sens Environ.

[31] Friberg M, Zhai X, Holmes H, Chang HH, Strickland MJ, Sarnat SE, Tolbert PE, Russell AG, Mulholland JA (2016). Method for fusing observational data and chemical transport model simulations to estimate spatiotemporally resolved ambient air pollution. Environ Sci Technol.

[32] Szpiro AA, Paciorek CJ (2013). Measurement error in two-stage analyses, with application to air pollution epidemiology. Environmetrics.

