# Supplementary material for: A simulation study to quantify the impacts of exposure measurement error on air pollution health risk estimates in copollutant time-series models
Source: Environ Health. 2016 Nov 25;15:114. doi: 10.1186/s12940-016-0186-0 (PMC5123332; doi:10.1186/s12940-016-0186-0)
Supplement: Additional file 1: — Supplemental materials including supplemental text, figures, and tables, are available as an additional file. (PDF 392 kb) [file 12940_2016_186_MOESM1_ESM.pdf]

Supplemental Material

A simulation study to quantify the impacts of exposure measurement error on air pollution health risk estimates in copollutant time-series models

Kathie L. Dionisio[1]

Howard H. Chang[2]

Lisa K. Baxter[1]

[1]National Exposure Research Laboratory, U.S. Environmental Protection Agency, RTP, NC, USA

[2]Department of Biostatistics and Bioinformatics, Emory University, Atlanta, GA, USA

Corresponding Author:
Kathie L. Dionisio
U.S. EPA
109 T.W. Alexander Drive
Mail Code: E205-02
Research Triangle Park, NC 27709
Tel: 919-541-1321
dionisio.kathie@epa.gov

Table of Contents

Supplemental Text…………………………………………………………………………….3

Figure S1………………………………………………………………..……………4-6

Table S1……………………………………………………………………………………7

Table S2……………………………………………………………………………………8

Table S3……………………………………………………………………………………9

Table S4……………………………………………………………………………………10

Supplemental Text

Using two-sided t-tests, we compared $\widehat{RR}_{1,noisy}$ for the same main pollutant, when varying the copollutant. Though differences in attenuation of the main pollutant RR by copollutant are small in magnitude (often on the order of 10^{-3} – 10^{-5}), these differences are significant for $\delta_{population}$ when NO_x is the main pollutant (Table S2). When the absolute difference in RR is not significant, it indicates that the choice of copollutant does not impact the RR of the main pollutant. In contrast, for the sensitivity analysis with an assumed $RR_1 = 1.05$ and $RR_2 = 1.05$, the choice of co-pollutant matters when NO_x is the main pollutant for all types of measurement error, and for EC and O_3 for $\delta_{population}$ (Table S3).

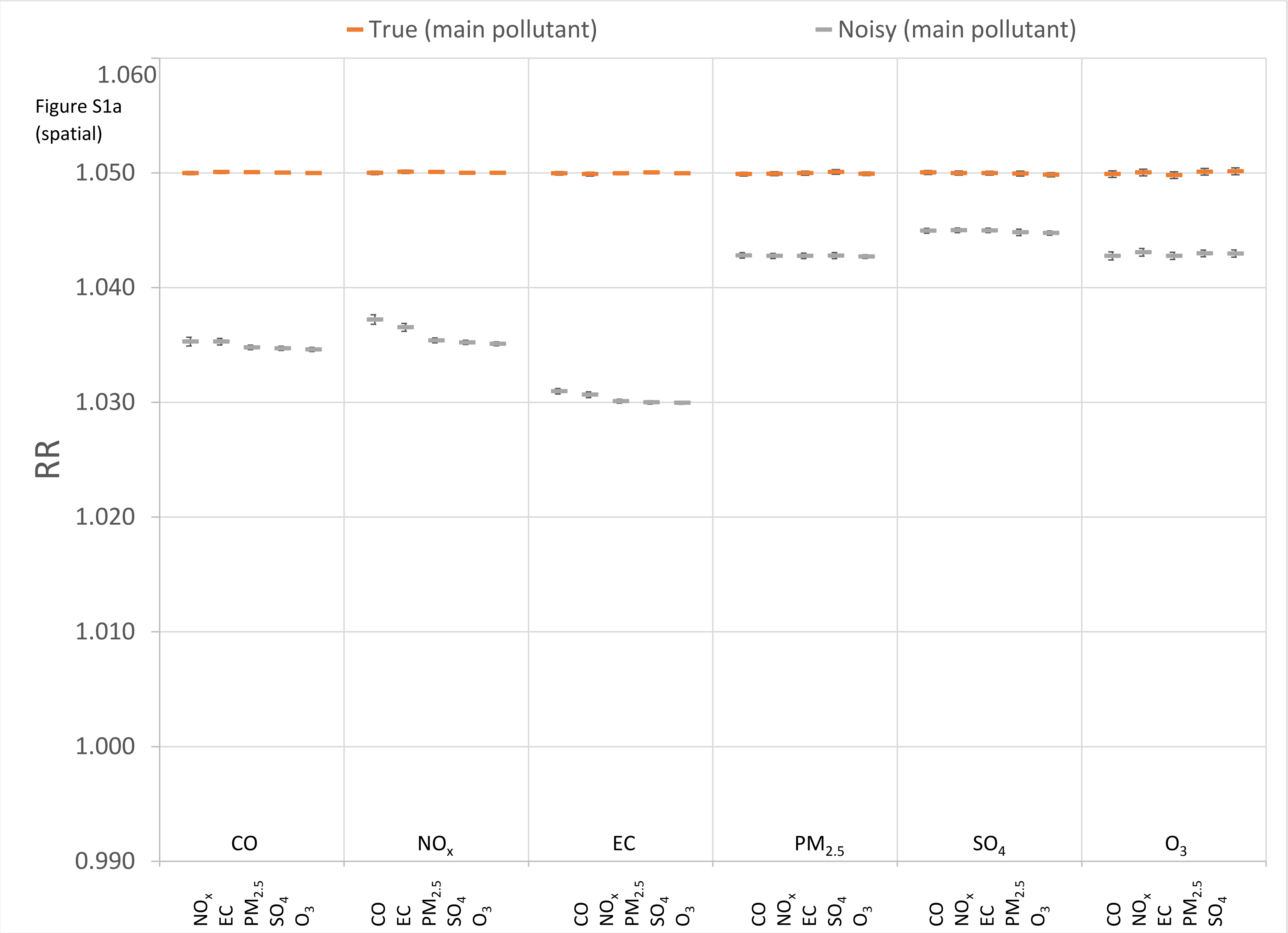

Figure S1: Attenuation of RR due to measurement error in a copollutant model (RR_1 = 1.05, RR_2 = 1.05). For x-axis labels, top row indicates the main pollutant (pollutant 1), bottom row indicates the copollutant (pollutant 2). Overall point estimates shown are the mean over 1,000 estimates; error bars indicate the 95th confidence interval for the 1,000 estimates (note that extremely narrow confidence intervals result in some non-visible error bars).

a) Spatial measurement error ($\delta_{spatial}$)

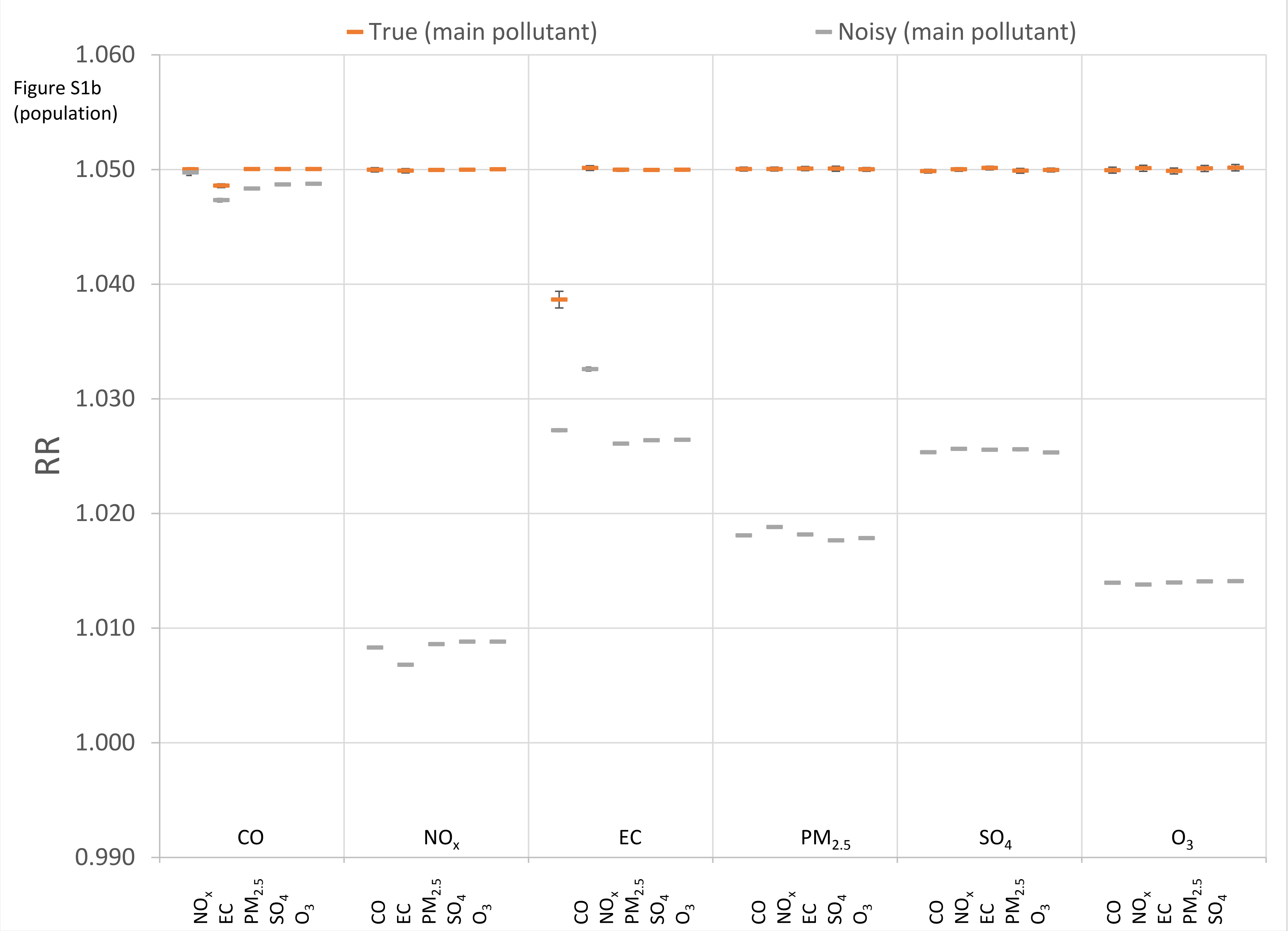

Figure S1: Attenuation of RR due to measurement error in a copollutant model ($RR_1 = 1.05$, $RR_2 = 1.05$). For x-axis labels, top row indicates the main pollutant (pollutant 1), bottom row indicates the copollutant (pollutant 2). Overall point estimates shown are the mean over 1,000 estimates; error bars indicate the 95th confidence interval for the 1,000 estimates (note that extremely narrow confidence intervals result in some non-visible error bars).

b) Population measurement error ($\delta_{population}$)

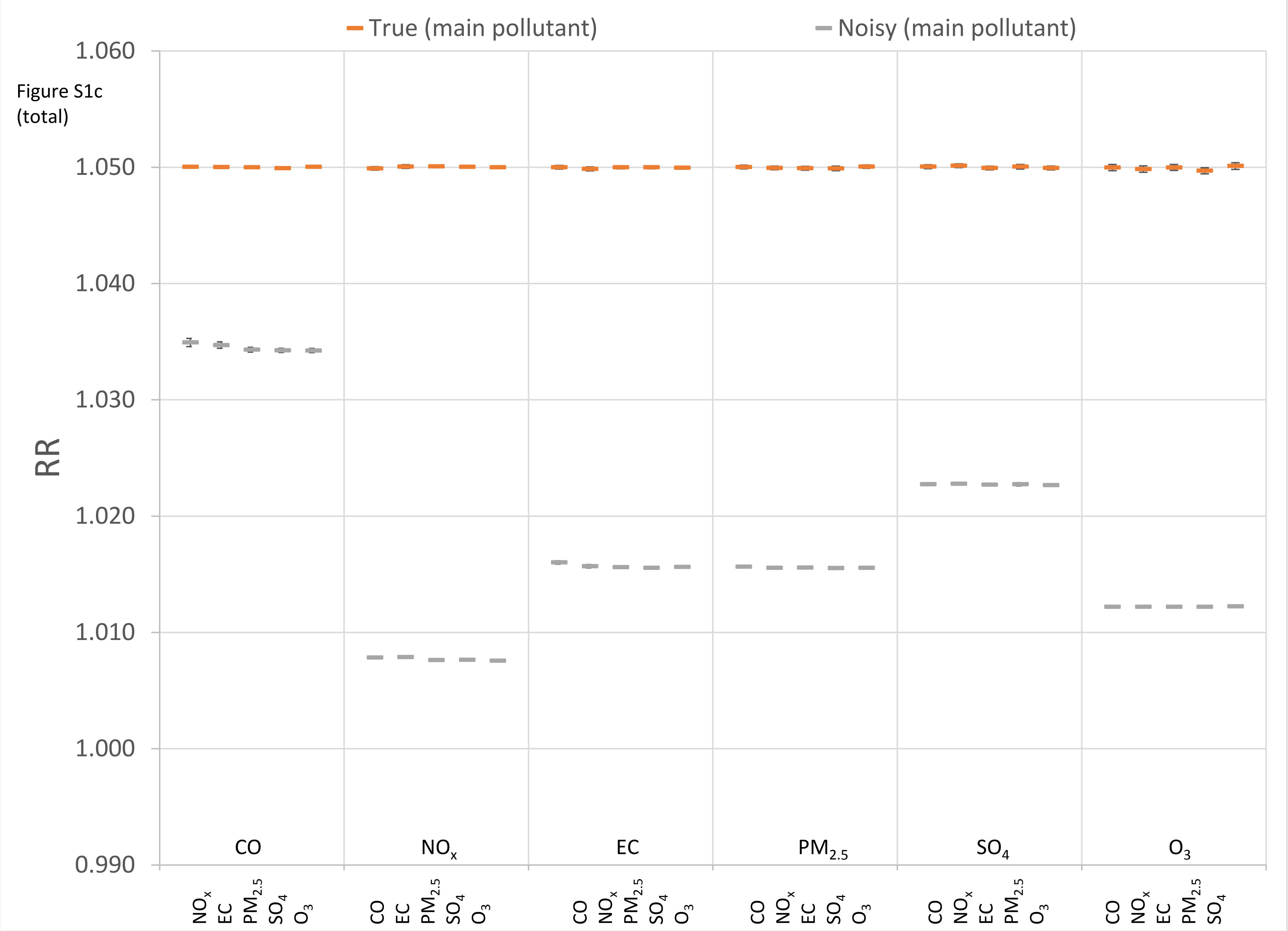

Figure S1: Attenuation of RR due to measurement error in a copollutant model (RR_1 = 1.05, RR_2 = 1.05). For x-axis labels, top row indicates the main pollutant (pollutant 1), bottom row indicates the copollutant (pollutant 2). Overall point estimates shown are the mean over 1,000 estimates; error bars indicate the 95th confidence interval for the 1,000 estimates (note that extremely narrow confidence intervals result in some non-visible error bars).

c) Total measurement error (δ_{total})

Table S1: Mean over all ZIP codes of additive (θ_1) and multiplicative (θ_2) bias, and of standard deviation (SD) of the residuals, for each pollutant, each measurement error type.

		**CO**	**NO_x**	**O_3**	**$PM_{2.5}$**	**SO_4**	**EC**
**Spatial**	θ_1 ± SD	0.1508 ± 0.0753	0.0236 ± 0.0207	0.0057 ± 0.0034	0.8846 ± 0.7505	0.3984 ± 0.0962	0.4385 ± 0.2110
	θ_2 ± SD	0.5703 ± 0.4338	0.5716 ± 0.4716	0.8183 ± 0.1115	0.8349 ± 0.0586	0.8743 ± 0.0644	0.5156 ± 0.2804
	SD of residuals ± SD	0.2854 ± 0.2362	0.0342 ± 0.0284	0.0048 ± 0.0014	3.4541 ± 0.6665	0.9698 ± 0.1062	0.6031 ± 0.4031
**Population**	θ_1 ± SD	0.0004 ± 0.0051	0.0014 ± 0.0013	0.0000 ± 0.0002	0.8408 ± 0.2096	0.0338 ± 0.0231	0.0800 ± 0.0390
	θ_2 ± SD	0.9996 ± 0.0597	0.2336 ± 0.0452	0.2892 ± 0.0119	0.3700 ± 0.0420	0.5134 ± 0.0364	0.5541 ± 0.0341
	SD of residuals ± SD	0.0099 ± 0.0154	0.0041 ± 0.0022	0.0015 ± 0.0001	0.9021 ± 0.1323	0.3009 ± 0.0333	0.0938 ± 0.0533
**Total**	θ_1 ± SD	0.1509 ± 0.0678	0.0059 ± 0.0045	0.0016 ± 0.0009	1.1143 ± 0.3471	0.2420 ± 0.0464	0.3402 ± 0.1481
	θ_2 ± SD	0.5644 ± 0.4210	0.1285 ± 0.0873	0.2376 ± 0.0272	0.3113 ± 0.0354	0.4495 ± 0.0485	0.2710 ± 0.1466
	SD of residuals ± SD	0.2825 ± 0.2280	0.0079 ± 0.0050	0.0019 ± 0.0002	1.5513 ± 0.2151	0.5865 ± 0.0700	0.3557 ± 0.2253

Table S2: Median over all ZIP codes of the correlation of measurement error covariance for each pollutant, each measurement error type.

		CO	NO_x	O_3	$PM_{2.5}$	SO_4	EC
**Spatial**	CO	1.00					
	NO_x	0.84	1.00				
	O_3	0.04	0.05	1.00			
	$PM_{2.5}$	0.18	0.32	0.12	1.00		
	SO_4	0.06	0.06	0.05	0.10	1.00	
	EC	0.68	0.82	0.03	0.34	0.15	1.00
**Population**	CO	1.00					
	NO_x	0.38	1.00				
	O_3	0.07	0.26	1.00			
	$PM_{2.5}$	0.05	0.36	0.47	1.00		
	SO_4	-0.07	0.13	0.30	0.76	1.00	
	EC	0.05	0.25	0.20	0.59	0.56	1.00
**Total**	CO	1.00					
	NO_x	0.72	1.00				
	O_3	-0.02	0.11	1.00			
	$PM_{2.5}$	0.08	0.25	0.23	1.00		
	SO_4	-0.02	0.05	0.12	0.28	1.00	
	EC	0.67	0.72	0.02	0.31	0.09	1.00

Table S3: Absolute difference in $RR_{1,noisy}$ for the same main pollutant, across different co-pollutants. * indicates pollutant pairs with a significant difference ($p<0.05$) in RR greater than 0.00. Outer labels on the x- and y-axis indicate the main pollutant (pollutant 1), inner labels indicate the co-pollutant (pollutant 2). For $RR_1 = 1.05$, $RR_2 = 1$.

$\delta_{spatial}$

		CO				
		NO_x	EC	$PM_{2.5}$	SO_4	O_3
CO	NO_x					
	EC	0.00				
	$PM_{2.5}$	0.00	0.00			
	SO_4	0.00	0.00	0.00		
	O_3	0.00	0.00	0.00	0.00	
		**NO_x**				
		CO	EC	$PM_{2.5}$	SO_4	O_3
NO_x	CO					
	EC	0.00				
	$PM_{2.5}$	0.00	0.01			
	SO_4	0.00	0.01	0.00		
	O_3	0.00	0.01	0.00	0.00	
		**EC**				
		CO	NO_x	$PM_{2.5}$	SO_4	O_3
EC	CO					
	NO_x	0.00				
	$PM_{2.5}$	0.00	0.00			
	SO_4	0.00	0.00	0.00		
	O_3	0.00	0.00	0.00	0.00	
		**$PM_{2.5}$**				
		CO	NO_x	EC	SO_4	O_3
$PM_{2.5}$	CO					
	NO_x	0.00				
	EC	0.00	0.00			
	SO_4	0.00	0.00	0.00		
	O_3	0.00	0.00	0.00	0.00	
		**SO_4**				
		CO	NO_x	EC	$PM_{2.5}$	O_3
SO_4	CO					
	NO_x	0.00				
	EC	0.00	0.00			
	$PM_{2.5}$	0.00	0.00	0.00		
	O_3	0.00	0.00	0.00	0.00	
		**O_3**				
		CO	NO_x	EC	$PM_{2.5}$	SO_4
O_3	CO					
	NO_x	0.02				
	EC	0.03	0.05			
	$PM_{2.5}$	0.05	0.07*	0.03		
	SO_4	0.01	0.01	0.04	0.07*	

$\delta_{population}$

		CO				
		NO_x	EC	$PM_{2.5}$	SO_4	O_3
CO	NO_x					
	EC	0.00				
	$PM_{2.5}$	0.00	0.00			
	SO_4	0.00	0.00	0.00		
	O_3	0.00	0.00	0.00	0.00	
		**NO_x**				
		CO	EC	$PM_{2.5}$	SO_4	O_3
NO_x	CO					
	EC	0.18*				
	$PM_{2.5}$	0.01*	0.19*			
	SO_4	0.03*	0.21*	0.02*		
	O_3	0.03*	0.21*	0.02*	0.01*	
		**EC**				
		CO	NO_x	$PM_{2.5}$	SO_4	O_3
EC	CO					
	NO_x	0.00				
	$PM_{2.5}$	0.00	0.00			
	SO_4	0.00	0.00	0.00		
	O_3	0.00	0.00	0.00	0.00	
		**$PM_{2.5}$**				
		CO	NO_x	EC	SO_4	O_3
$PM_{2.5}$	CO					
	NO_x	0.00				
	EC	0.00	0.00			
	SO_4	0.00	0.00	0.00		
	O_3	0.00	0.00	0.00	0.00	
		**SO_4**				
		CO	NO_x	EC	$PM_{2.5}$	O_3
SO_4	CO					
	NO_x	0.00				
	EC	0.00	0.00			
	$PM_{2.5}$	0.00	0.00	0.00		
	O_3	0.00	0.00	0.00	0.00	
		**O_3**				
		CO	NO_x	EC	$PM_{2.5}$	SO_4
O_3	CO					
	NO_x	0.05				
	EC	0.05	0.00			
	$PM_{2.5}$	0.06	0.01	0.01		
	SO_4	0.05	0.01	0.01	0.01	

δ_{total}

		CO				
		NO_x	EC	$PM_{2.5}$	SO_4	O_3
CO	NO_x					
	EC	0.00				
	$PM_{2.5}$	0.00	0.00			
	SO_4	0.00	0.00	0.00		
	O_3	0.00	0.00	0.00	0.00	
		**NO_x**				
		CO	EC	$PM_{2.5}$	SO_4	O_3
NO_x	CO					
	EC	0.00				
	$PM_{2.5}$	0.01	0.01			
	SO_4	0.01	0.00	0.00		
	O_3	0.01	0.01	0.00	0.00	
		**EC**				
		CO	NO_x	$PM_{2.5}$	SO_4	O_3
EC	CO					
	NO_x	0.00				
	$PM_{2.5}$	0.00	0.00			
	SO_4	0.00	0.00	0.00		
	O_3	0.00	0.00	0.00	0.00	
		**$PM_{2.5}$**				
		CO	NO_x	EC	SO_4	O_3
$PM_{2.5}$	CO					
	NO_x	0.00				
	EC	0.00	0.00			
	SO_4	0.00	0.00	0.00		
	O_3	0.00	0.00	0.00	0.00	
		**SO_4**				
		CO	NO_x	EC	$PM_{2.5}$	O_3
SO_4	CO					
	NO_x	0.00				
	EC	0.00	0.00			
	$PM_{2.5}$	0.00	0.00	0.00		
	O_3	0.00	0.00	0.00	0.00	
		**O_3**				
		CO	NO_x	EC	$PM_{2.5}$	SO_4
O_3	CO					
	NO_x	0.02				
	EC	0.02	0.01			
	$PM_{2.5}$	0.01	0.02	0.03		
	SO_4	0.03	0.02	0.01	0.04	

Table S4: Absolute difference in $RR_{1,noisy}$ for the same main pollutant, across different co-pollutants. * indicates pollutant pairs with a significant difference ($p<0.05$) in RR greater than 0.00. Outer labels on the x- and y-axis indicate the main pollutant (pollutant 1), inner labels indicate the co-pollutant (pollutant 2). For $RR_1 = 1.05$, $RR_2 = 1.05$.

		$\delta_{spatial}$					$\delta_{population}$					δ_{total}				
		CO					CO					CO				
		NO_x	EC	$PM_{2.5}$	SO_4	O_3	NO_x	EC	$PM_{2.5}$	SO_4	O_3	NO_x	EC	$PM_{2.5}$	SO_4	O_3
CO	NO_x															
	EC	0.00					0.01*					0.00				
	$PM_{2.5}$	0.00	0.00				0.00	0.00				0.00	0.00			
	SO_4	0.00	0.00	0.00			0.00	0.00	0.00			0.00	0.00	0.00		
	O_3	0.00	0.00	0.00	0.00		0.00	0.00	0.00	0.00		0.00	0.00	0.00	0.00	

		NO_x					NO_x					NO_x				
		CO	EC	$PM_{2.5}$	SO_4	O_3	CO	EC	$PM_{2.5}$	SO_4	O_3	CO	EC	$PM_{2.5}$	SO_4	O_3
NO_x	CO															
	EC	0.03*					0.06*					0.00				
	$PM_{2.5}$	0.07*	0.04*				0.04*	0.10*				0.04*	0.04*			
	SO_4	0.08*	0.05*	0.01			0.07*	0.13*	0.03*			0.04*	0.04*	0.00		
	O_3	0.08*	0.05*	0.01*	0.00		0.07*	0.13*	0.03*	0.00		0.05*	0.05*	0.01*	0.01*	

		EC					EC					EC				
		CO	NO_x	$PM_{2.5}$	SO_4	O_3	CO	NO_x	$PM_{2.5}$	SO_4	O_3	CO	NO_x	$PM_{2.5}$	SO_4	O_3
EC	CO															
	NO_x	0.00					0.01*					0.00				
	$PM_{2.5}$	0.00	0.00				0.00	0.01*				0.00	0.00			
	SO_4	0.00	0.00	0.00			0.00	0.01*	0.00			0.00	0.00	0.00		
	O_3	0.00	0.00	0.00	0.00		0.00	0.01*	0.00	0.00		0.00	0.00	0.00	0.00	

		$PM_{2.5}$					$PM_{2.5}$					$PM_{2.5}$				
		CO	NO_x	EC	SO_4	O_3	CO	NO_x	EC	SO_4	O_3	CO	NO_x	EC	SO_4	O_3
$PM_{2.5}$	CO															
	NO_x	0.00					0.00					0.00				
	EC	0.00	0.00				0.00	0.00				0.00	0.00			
	SO_4	0.00	0.00	0.00			0.00	0.00	0.00			0.00	0.00	0.00		
	O_3	0.00	0.00	0.00	0.00		0.00	0.00	0.00	0.00		0.00	0.00	0.00	0.00	

		SO_4					SO_4					SO_4				
		CO	NO_x	EC	$PM_{2.5}$	O_3	CO	NO_x	EC	$PM_{2.5}$	O_3	CO	NO_x	EC	$PM_{2.5}$	O_3
SO_4	CO															
	NO_x	0.00					0.00					0.00				
	EC	0.00	0.00				0.00	0.00				0.00	0.00			
	$PM_{2.5}$	0.00	0.00	0.00			0.00	0.00	0.00			0.00	0.00	0.00		
	O_3	0.00	0.00	0.00	0.00		0.00	0.00	0.00	0.00		0.00	0.00	0.00	0.00	

		O_3					O_3					O_3				
		CO	NO_x	EC	$PM_{2.5}$	SO_4	CO	NO_x	EC	$PM_{2.5}$	SO_4	CO	NO_x	EC	$PM_{2.5}$	SO_4
O_3	CO															
	NO_x	0.04					0.08*					0.01				
	EC	0.02	0.05				0.02	0.10*				0.02	0.01			
	$PM_{2.5}$	0.01	0.03	0.02			0.08*	0.17*	0.07			0.02	0.01	0.00		
	SO_4	0.01	0.02	0.03	0.01		0.10*	0.18*	0.09*	0.02		0.01	0.01	0.02	0.03	
